# Adherence to central venous catheter maintenance bundle in an intensive care unit

**DOI:** 10.1590/1980-220X-REEUSP-2022-0077en

**Published:** 2022-09-23

**Authors:** Amanda Inocencio de Quadros, Janislei Giseli Dorociaki Stocco, Cristiane Cristoff, Camila Bonfim de Alcantara, Adriano Marçal Pimenta, Bruna Giane Saidelles Machado

**Affiliations:** 1 Universidade Federal do Paraná, Curitiba, PR, Brazil.; 2 Universidade Federal do Paraná, Complexo do Hospital de Clínicas, Curitiba, PR, Brazil.

**Keywords:** Intensive Care Units, Central Venous Catheters, Catheter-Related Infections, Patient Care Bundles, Unidades de Cuidados Intensivos, Catéteres Venosos Centrales, Infecciones Relacionadas con Catéteres, Paquetes de Atención al Paciente, Unidades de Terapia Intensiva, Cateteres Venosos Centrais, Infecções Relacionadas a Cateter, Pacotes de Assistência ao Paciente

## Abstract

**Objective:**

To check adherence to the Central Venous Catheter maintenance bundle in an Intensive Care Unit, after an educational intervention to the professionals who provide care to patients using this catheter.

**Method:**

Descriptive-exploratory study, carried out in two stages: stage 1 – educational intervention and stage 2 – verification/observation of adherence. Data were organized in the *Microsoft Excel*® and analyzed through the Stata®.

**Results:**

Sixty three workers participated in stage 1 and 44 in stage 2. The sample consisted of 64 observation opportunities. Among the domains observed, the recording of indication of permanence had an 8% compliance rate; aseptic technique in catheter handling, 3%; maintenance of the infusion system, 15%; and care with the central venous catheter dressing, 17%. The domains represent unwanted care according to the Positivity Index for assessing the quality of care.

**Conclusion:**

The findings show the need for discussions, training, and investments in constant strategies for the prevention of primary bloodstream infections related to the central venous catheter.

## RESUMO

**Objetivo:**

Verificar a adesão ao *bundle* de manutenção do Cateter Venoso Central em uma Unidade de Terapia Intensiva, após intervenção educativa aos profissionais que realizam o cuidado aos pacientes em uso desse cateter.

**Method:**

Estudo descritivo-exploratório, realizado em duas fases, fase 1 – intervenção educativa, e fase 2 – verificação/observação da adesão. Os dados foram organizados no *Microsoft Excel* ® e analisados por meio do *Stata*®.

**Resultados:**

Participaram da fase 1 63 profissionais e da fase 2, 44. A amostra foi constituída de 64 oportunidades de observações. Entre os domínios observados, o registro de indicação de permanência apresentou 8% de taxa de conformidade; a técnica asséptica no manuseio do cateter, 3%; a manutenção do sistema de infusão, 15%; e os cuidados com o curativo do cateter venoso central, 17%. Os domínios representam uma assistência indesejada, conforme o Índice de Positividade de avaliação da qualidade da assistência.

**Conclusion:**

Os achados mostram a necessidade de discussões, treinamentos e investimentos em estratégias constantes para a prevenção de infecções primárias de corrente sanguínea relacionadas ao cateter venoso central.

## RESUMEN

**Objetivo:**

Averiguar la adhesión al *bundle* de manutención del Catéter Venoso Central en una unidad de terapia intensiva, tras intervención educativa a los profesionales que realizan el cuidado a los pacientes en uso de este catéter.

**Método:**

Estudio descriptivo exploratorio, realizado en dos etapas: Etapa 1 – intervención educativa y etapa 2 – averiguación/observación de la adhesión. Los datos fueron organizados en el *Microsoft Excel*® y analizados a través del *Stata*®.

**Resultados:**

Participaron de la etapa 1 63 profesionales y de la etapa 2, 44. La muestra fue constituida de 64 oportunidades de observaciones. Entre los dominios observados, el registro de indicación de permanencia presentó el 8% de tasa de conformidad, la técnica aséptica en el manejo del catéter, 3%, la manutención del sistema de infusión, 15% y los cuidados con el vendaje del catéter venoso central, 17%. Los dominios representan una asistencia no deseada de acuerdo con el Índice de Positividad de evaluación de la calidad de la asistencia.

**Conclusión:**

Los hallazgos demuestran la necesidad de debates, entrenamientos e inversiones en estrategias constantes para la prevención de infecciones primarias de corriente sanguínea relacionadas al catéter venoso central.

## INTRODUCTION

Health care has as main objective to bring significant improvements to patients. However, the risk during its execution is recognized and can expose users to different outcomes and consequences, generating physical, social, and economic damages; thus, there has been much discussion over the years on patient safety in hospital environments^(1)^.

In 2009, the World Health Organization (WHO) conceptualized patient safety as the reduction, to a considerable minimum, of the risk of unnecessary harm associated with health care. Among the concerns in the field of patient safety is the reduction in the number of healthcare-associated infections (HAIs)^(1)^.

Among the HAIs with the highest occurrence, primary bloodstream infection (PBSI) is highlighted, due to its relation to the use of central venous catheters (CVC). PBSI is associated with a high mortality rate, increased length of stay, and increased patient care-related costs^(2)^.

In Latin America, the incidence of CVC-associated PBSI was estimated at 7 episodes per 1000 catheter-day, while in some studies carried out in Europe and the United States of America, 2–3 episodes/1000 catheter-day were reported^(3)^. In Brazil, according to the bulletin of the Brazilian Health Regulatory Agency, the incidence of CVC-associated PBSI in adult ICUs in 2016 was 4.6 infections per 1,000 CVC/day^(4)^.

PBSI is associated with an increase in length of hospital stay by approximately 10 to 20 days, with a cost of US$30,000.00 per patient^(5)^. Estimates from a meta-analysis showed that the annual costs generated by the HAIs were US$ 9.8 billion. Among the HAIs, the bloodstream ones had a cost per treatment of US$ 45,814.00; when associated with a resistant microorganism, generated an increase in cost to US$ 58,614.00^(6)^. Another meta-analysis that sought to identify mortality attributable to PBSI resulted in an odds ratio of PBSI-associated deaths of 2.75 (CI 1.86 – 4.07) with most of them in patients admitted to the Intensive Care Unit (ICU)^(7)^.

Given the relevance of CVC-related PBSIs and their implications for patients and institutions, the development of strategies to reduce modifiable factors is required.

One of the strategies to reduce PBSI is the adoption of measures in the form of intervention packages, described as bundles. At the CVC bundle, hand hygiene, use of maximum precautionary barriers, skin antisepsis with chlorhexidine gluconate are required, as well as the selection of the insertion site, avoiding the use of the femoral vein due to the possibility of device contamination. After CVC insertion, hand hygiene before handling the device, rubbing of the connectors and catheter connection with 70% alcohol, dressing care, and daily check for the permanence of the catheter are indicated^(2,8)^.

For the care mentioned above to be incorporated into care practice and help in the reduction and prevention of PBSI, multidisciplinary team training and sensitization are required, since non-adherence to the bundles can compromise the quality of care and patient safety^(2,8)^.

In addition, monitoring adherence to PBSI prevention measures becomes an important strategy for pointing out gaps and providing support for the development of improvements in care practice^(2,8)^.

Therefore, the objective of this study was to check adherence to the Central Venous Catheter maintenance bundle in an Intensive Care Unit, after an educational intervention to the professionals who provide care to patients using this catheter.

## METHOD

### Desig of Study

This is a descriptive-exploratory study, with a quantitative approach, involving the observation of the adherence of the nursing team to the CVC maintenance bundle.

### Local

The study was carried out in an ICU of a public teaching hospital in Curitiba-PR. The unit provides health care to patients over 18 years of age who require intensive care. During the data collection period, the unit had 16 beds, with 65 nursing professionals, of which 19 were nurses and 46 were technicians. It should be noted that the unit did not have the bundle established in routines and protocols.

### Selection Criteria

The eligibility criteria for participation in the study were professionals from the care team (nurses and nursing technicians) working in the unit, who handled CVC and participated in Stage 1 – educational intervention. Professionals not found due to vacation or sick leave during data collection were excluded from the study.

### Sample Definition

The sample was non-probabilistic, and was for convenience, corresponding to the opportunities of direct observation of the practices in the three work shifts (morning, afternoon, and night) from July to August 2021.

### Data Collection

The study took place in two distinct stages: stage 1 – educational intervention and stage 2 – verification/observation of adherence to the CVC maintenance bundle. In the first stage, all nursing professionals working in the service were verbally invited to participate in the study, and they were also asked to participate in an educational intervention on the CVC maintenance bundle. Participation in the educational intervention was provided to all professionals who were interested. However, this activity was considered mandatory for participants to become eligible for the second stage of the study.

The educational intervention took place from March to June 2021, and was offered in all shifts (morning, afternoon, and night) to facilitate the participation of the largest number of professionals.

The intervention took place through the realistic simulation method with the use of a mannequin with a CVC inserted and available for the handling and administration of fluids, fictitious prescription, and the scenario of an ICU bed. The intervention was carried out in a reserved place close to the unit’s facilities and during the professionals’ working hours, to facilitate and encourage participation.

Participants were divided into pairs or trios, with the professional who would perform the simulation of care that composes PBSI prevention measures in CVC maintenance being defined among the members, with the other participants being observers, with the function of scoring items performed correctly and neglected items. After the end, a joint discussion was held among professionals and researchers comparing the practices performed and those recommended in the bundle.

In the second stage of verification/observation of adherence to the CVC maintenance bundle, the study population consisted of the nursing team performing the CVC-related care practices in the morning, afternoon, and night shifts. The workers’ participation was established after they signed the Free and Informed Consent Form (FICF), after receiving explanation on the study from the researcher.

In the first moment, the variables of the sample sociodemographic characterization were obtained, namely: sex, age, profession, and experience in the ICU.

For data collection and recording, the bundle prepared with guidelines from the Institute for Healthcare Improvement (IHI)^(9)^ was used, provided by the Institutional Development Support Program of the Brazilian Public Healthcare System (*PROADI-SUS*). The instrument was converted into a checklist about adherence to the CVC maintenance bundle. That bundle presents direct questions such as “yes” for adequate, “no” for not adequate, and “does not apply” for not observed. The instrument is divided into 4 domains: 1) Registry indicating permanence of CVC: registration of the insertion date on the medical record; the reason for CVC permanence; 2) Adherence to aseptic technique in handling the catheter: hand hygiene (with 70% alcohol or soap and water) before and after handling the device; hub disinfection (performing the disinfection through mechanical rubbing of connections, vascular access tubes, three-ways, and connectors with 70% alcoholic antiseptic solution for 5–15 seconds, before administration or collection of venous fluids); replacement of the occluding cap after manipulation; 3) Maintenance of the infusion system in accordance with current recommendations: hand hygiene (with 70% alcohol or soap and water) before and after handling the catheter; exchange of equipment every 96 hours for continuous infusions and 24 hours for intermittent infusions; equipment and complementary device of Total Parenteral Nutrition (TPN) for each bag; propofol equipment and complementary device every 12 hours; hemodynamic monitoring equipment every 96 hours; flushing with 5 to 10 ml of saline solution (0.9% sodium chloride) between and after medications; 4) Adherence to dressing care: hand hygiene (with 70% alcohol or soap and water) before and after handling the catheter; dressing change with aseptic technique and use of 5% alcoholic chlorhexidine; sterile dressing with date identification; frequency of dressing change, with conventional dressing (gauze and sterile adhesive tape) being changed every 48 hours or earlier if there is dirt and dressings with a sterile transparent cover every 7 days or earlier if there is dirt or poor adherence to the skin.

Before data collection, the researchers were trained to reduce bias during data measurement, which consisted of a theoretical-practical step on the items of the maintenance bundle that would be evaluated, followed by a step of double observation by the principal investigator and other researchers, comparison to what was observed, and discussion of the results obtained, to assess the reliability of the recorded data. The agreement between the two observers was calculated using the *kappa* coefficient with 92.5% agreement, and K of *Cohen* of 0.85 indicating almost complete agreement^(10)^.

Casuistry corresponded to opportunities for evaluating the listed practices performed by nurses and nursing technicians, which were observed during the CVC handling in adult patients admitted to the ICU. Among the items that make up the CVC maintenance bundle domains, the number of observations is defined by the development of this item by the professional during the opportunity observed by the researcher, and some professionals were observed more than once.

It should be noted that the observations were carried out in the three shifts and considering the even and odd nights, to cover all professionals. Data collection through direct observation of professional practice took place over a period of 60 days interspersed during the week. As for the time allocated to this stage, it should be noted that during daytime, the observation time ranged from six to four hours, according to the availability of the researchers and considering the unit’s medication schedule, so as to prioritize observation in the periods when there is a greater chance of manipulation of the devices due to the need to administer drugs or other solutions. At night, the observation was concentrated between 7 pm and 10pm, for both nights, odd and even, because it is a period of greater number of CVC manipulations due to the standardized medication schedule in the unit.

## Data Analysis

For each CVC manipulation, compliance or non-compliance with the items to prevent catheter-related infection was observed.

For global adherence, adherence to the domain was considered when the worker adhered to all the items that make up that observed domain, considering that the bundle refers to the set of measures to prevent bloodstream infection. For example, for hand hygiene, compliance was considered when the worker performed hand hygiene before and after the procedure using water and antiseptic soap or 70% alcohol, and non-compliance when the worker did not sanitize his/her hands or did it only in one moment.

To establish the expected compliance of the evaluated practices related to the CVC maintenance bundle, the Positivity Index (PI) proposed by Carter was used^(11)^, which was adopted in other studies evaluating the quality of care, where 100% adherence represents desirable care; from 90 to 99%, adequate assistance; from 80 to 89%, safe assistance; from 70 to 79%, a borderline assistance and less than 70%, an unwanted assistance.

Data were organized in electronic spreadsheets in the software Microsoft Office Excel 2013 and analyzed with the aid of the statistical software Data Analysis and Statistical Software (Stata). The adequacy percentages by professional category and work shift were evaluated for each item.

## Ethical Aspects

The workers’ participation was established after they signed the Free and Informed Consent Form (FICF), after receiving explanation on the study from the researcher.

The study was developed after approval by the Human Research Ethics Committee under opinion n. 4.161.849 of 2020, in accordance with Resolution no. 466/12, of the National Health Council.

## RESULTS

Among the 65 professionals of the nursing team, 63 (97%) participated in stage 1 – educational intervention on the CVC maintenance bundle. In stage 2 – observation of adherence to the CVC maintenance bundle, 44 professionals participated, resulting in 70% of the nursing team.

Among the 44 professionals who agreed to participate in the observation stage of adherence to the CVC maintenance bundle, most were nursing technicians (n = 31, 70%), women (n = 32, 73%), aged between 30 and 39 years (n = 27, 62%). Regarding professional experience, most participants stated that they had some experience in the ICU (n = 28, 64%), and the reported time was one year providing assistance in the intensive care department (n = 19, 43 %).

The sample consisted of 64 observation opportunities, since the same professional was observed on more than one opportunity. The observations are distributed simultaneously in 21 in the morning shift, 22 in the afternoon shift, 11 in the even night and 10 in the odd night team.

The CVC maintenance bundle corresponds to the four domains: recording of indication of CVC permanence, adherence to aseptic technique in catheter handling, maintenance of the infusion system and adherence to dressing care.

In Table 1, the variables that were verified/observed related to the domains that make up the CVC maintenance bundle, listing adherence or nonadherence per domain item.

**Table 1.** Domains of central venous catheters bundle verified/observed in an Intensive Care Unit – Curitiba, PR, Brazil, 2021.

Variables	Adherence		Nonadherence		Total	
	n	%*	n	%*	n	%*
**Domain 1 – Recording of indication of CVC permanence**						
Record of length of stay
	32	50%	32	50%	64	100%
Recording of indication of CVC
	5	8%	59	92%	64	100%
**Domain 2 – Adherence to aseptic technique in catheter handling**						
Hand hygiene before device handling
	15	36%	27	64%	42	100%
Hand hygiene after device handling
	11	28%	31	72%	42	100%
Disinfection of hub or valved connectors/occluders with 70% alcohol
	31	74%	11	26%	42	100%
Replacement of the occluder cap after opening the system
	2	67%	1	33%	3	100%
**Domain 3 – Maintenance of the infusion system**						
Hand hygiene before device handling
	12	32%	25	68%	37	100%
Hand hygiene after device handling
	13	38%	23	62%	37	100%
Replacement of equipment/validity
	42	67%	21	33%	63	100%
Flush between infusions or blood draws
	17	49%	18	51%	35	100%
**Domain 4 – Adherence to dressing care**						
Hand hygiene before device handling
	5	42%	7	58%	12	100%
Hand hygiene after device handling
	9	75%	3	25%	12	100%
Dressing change with aseptic technique
	8	67%	4	33%	12	100%
Sterile dressing with date identification
	59	92%	5	8%	64	100%
Frequency of dressing change
	52	81%	12	19%	64	100%

Note: The percentages were calculated considering the number of times in which there was the opportunity to observe each action/practice being performed.

In the domains presented, the item with the highest adherence refers to the adequate identification of the CVC dressing (92%), followed by the item of frequency of dressing change (81%), considering that they were dated and in accordance with the expiration date. It should be noted that, in the institution, the performance of the CVC dressing is defined in the protocol as an attribution of the nurse.

For each domain involving the manipulation of the catheter, the performance of hand hygiene before and after manipulation was evaluated. Among the three domains observed, the one with the highest adherence to hand hygiene before catheter handling was the domain of adherence to dressing care, with a percentile of 42%.

The item with the lowest adherence was the indication record of CVC permanence with a 92% percentile of non-adherence.

In the domain of adherence to aseptic technique in catheter handling, the item to change the occluder cap after opening the system had only 3 observation opportunities, since most CVCs had valved occluders and are not applicable to this item in the study.

In Table 2, the quality of care was evaluated according to the global adherence of the domains making up the CVC maintenance bundle.

**Table 2.** Assessment of the quality of assistance by domain of central venous catheter maintenance bundle, according to the Positivity Index (PI) – Curitiba, PR, Brazil, 2021.

Variables	Positivity Index (%)	Quality of assistance
**Domains**			
Indication record of CVC permanence time	8%	Unwanted assistance
Adherence to aseptic technique in catheter handling	3%	Unwanted assistance
Maintenance of the infusion system	15%	Unwanted assistance
Adherence to dressing care	17%	Unwanted assistance

Based on the quality of care criteria according to the positivity index, all domains suggest unwanted care.

The domain “adherence to dressing care” had the highest rate of positivity in the study (17%), but below the score to be considered as borderline assistance, in which values above 70% of PI should be obtained.

The domain with the lowest rate of positivity was “adherence to aseptic technique when handling the catheter” (3%), consisting of hand hygiene items before and after handling the catheter, disinfection of the hub or connectors with 70% alcohol and replacement of the occluding cap (when in use) after opening the system.

## DISCUSSION

The sociodemographic characterization of this study pointed to a greater number of women, in line with other studies that also show a significant percentage of women in the nursing team. This predominance of women in the profession reflects sociocultural and historical values, where care was strongly associated and attributed to women^(12)^.

Still regarding the characterization of the sample, most professionals claimed to have some experience in the ICU, with this period of up to one year providing health care to critically ill patients. Patient safety can be little addressed in professional training and, when addressed, this is done only occasionally, with no in-depth reflections. Therefore, improvement in the training and qualification processes, regarding aspects related to CVC-associated PBSI, with a view to improving the quality of care, is required for professionals already in the job market^(13)^.

In domain 1 – indication record of catheter permanence time, it should be noted that in 100% of the observations made in this research, only 50% showed adherence to the item of recording CVC permanence time, and 8% to the indication record of CVC permanence. Recording the time and reason for CVC permanence allows its removal when there is no longer any clinical indication for its use. When the catheter is in place for more than 1–2 weeks, CVC-associated infection rates increase^(14)^.

The adequate record in the patient’s medical record reflects the quality of care, and can be one of the indicators of work productivity. Such records will serve as a basis for building improvements in care practices and implementing actions aimed at improving operational results^(14)^.

Domain 2 observed adherence to aseptic technique in handling the catheter, covering the following items: hand hygiene, hub disinfection, exchange of connectors. Regarding hand hygiene, this is the most effective practice in preventing healthcare-associated infections. Despite its importance, adherence to this practice remains low in health services, as observed in this study. A systematic review that included 65 studies carried out in an ICU showed an average adherence rate of 40%, and an ideal percentage above 70% is considered for adherence^(15)^.

Among the strategies to improve adherence to hand hygiene is the use of alcohol-based handrub when in the absence of body fluids on the hands. Among the positive points of this practice are the quick action to reduce the microbial load on the skin, short time for cleaning, availability of material at the time of assistance, and no need for special infrastructure^(16)^.

It should be highlighted that there were few opportunities for observation regarding the replacement of the occluding cap, due to the frequent use of valved connectors. However, the health care-related infection prevention measures manual^(4)^ indicates the need to carefully monitor infection rates after the introduction of valved connectors. Hub disinfection showed an adherence of 74% of the observations; however, the positivity index was low in this domain. A study highlights that maintenance bundles activities that include hub disinfection were associated with reduced PBSI^(17)^.

Failure to disinfect the hub results in contamination and subsequent biofilm formation within the connectors and catheters, increasing the potential for infection of central catheters. A systematic review evaluating 140 studies on connector disinfection practices reports that adherence to disinfection is less than 10%, and it is essential that health establishments assume the responsibility for complying with the basic principles of asepsis, promoting education and involvement of the healthcare team front line that performs this care^(18)^.

In domain 3, infusion system maintenance, the present study showed adherence of 67% in the exchange of equipment and 15% in the flushing between infusions. According to the guidance of the Centers for Disease Control and Prevention (CDC)^(5)^, the exchange of equipment carried out between 72 and 96 hours reduces CVC contamination. In France, it is recommended that implanted catheters be flushed with 10 mL of sterile saline to avoid obstruction, which is a potential source of infection^(19)^. Data from this domain reinforce the need for strategies emphasizing adherence to this practice, to prevent CVC infections, since the PI demonstrated unwanted assistance.

The items that make up domain 4, adherence to dressing care, obtained the best adherence scores. However, they reflect unwanted assistance from the PI. The central venous access dressing is a way to protect the catheter insertion site against microorganisms that can colonize the ostium. The need for care with the maintenance and protection of the dressing is highlighted. They should not be wet and the use of waterproof cover is recommended, since it reduces the entry of moisture and contaminants into the catheter^(20)^.

In this study, the development of the educational intervention was through an activity in the form of realistic simulation, justified by the need for dynamic interventions for adherence to the listed items. However, as observed in a study, the development of such skills will not necessarily improve care practice, since people act according to their intentions and perceptions, reinforcing the need for team awareness raising^(21)^.

Effective multicenter interventions addressing CVC maintenance care have used several different modalities in training programs. Interventions included didactic lectures, self-study modules, checklists, simulations, online site-based training. The advantage of the online training course is that it does not require additional staff to give lectures and it provides study autonomy and flexibility to the worker^(22)^.

Another study reports that it used simulation-based training, face-to-face meetings, one-on-one educational sessions, and these were essential for the implementation of the bundles. The authors concluded that this type of approach was effective in implementing changes and improving outcomes, promoting teamwork, improving adherence, and providing the feedback^(23)^.

The development of capabilities allied to actions to implement the bundle has reduced the numbers of CVC-related PBSI. In Taiwan, a 10-month intervention study was performed in 5 ICUs (18,656 patients/day and 9,388 catheters/day), with a significant decrease in CVC-related PBSI rates from 1.65 per 1000 catheter/day to 0.65 per 1000 catheter/day^(24)^.

In a prospective US before-after intervention study conducted in two ICUs (1,141 patients and 3,784 catheters/day) over 11 months, the rate of CVC-related PBSI decreased from 5.02 to 1.60 per 1,000 catheters/day, after the implementation of the bundle^(25)^.

A study aiming to evaluate the knowledge and behavior of professionals in relation to recommended actions in bundles, for the prevention of CVC-associated PBSI, comments that for adherence to prevention measures, it is necessary to invest in updating and training, involving the team in the construction of an action plan after analyzing indicators^(26)^.

Considering the results obtained in the study, the supervision of the unit was communicated to highlight the need for educational interventions and to investigate the factors related to noncompliance by professionals of the recommendations of the CVC maintenance bundle that required attention. In light of this discussion, the implementation of the bundles in the care practice of professionals who work in unit studied was initiated.

As for the limitations of the study, the constant changes in the composition of the team can be highlighted, as well as the profile of patients hospitalized in the unit due to the pandemic scenario, hindering the stages of educational intervention and data collection. The sample size is also a bias in the study, as it limits the generalization of results, requiring further studies that include a greater number of professionals, greater opportunities for observations, and also other ICUs in the institution.

## CONCLUSION

The results showed a lower-than-expected positivity index in all the items that make up the bundle, evidencing an unwanted assistance with regard to care for CVC maintenance, also reflecting negatively on the prevention of PBSI.

Findings from the study demonstrate the importance of team discussions on the prevention of CVC-related primary bloodstream infections and the need for investments in continuing education and the development of motivational strategies, as well as the involvement of specific units such as that for Infection, Permanent Education and Patient Safety.

Training continuity for the care team after the implementation of the bundles, as well as continuing education programs, are essential for infection prevention and safety of the patient on CVC. Studies with more robust samples are recommended to investigate adherence and the effectiveness of implementing this strategy in Intensive Care Units.
